# Association between xerostomia, oral and general health, and obesity in adults. A cross-sectional pilot study

**DOI:** 10.4317/medoral.24731

**Published:** 2021-05-23

**Authors:** Alba Pérez-González, Juan A Suárez-Quintanilla, Eva Otero-Rey, Andrés Blanco-Carrión, Francisco J Gómez-García, Pilar Gándara-Vila, Benjamín Martín-Biedma, Mario Pérez-Sayáns

**Affiliations:** 1 Oral Medicine, Oral Surgery and Implan, Spaintology Unit (MedOralRes). Faculty of Medicine and Dentistry, Universidade de Santiago de Compostela, Spain; 2 Area of Human Anatomy and Embryology, Faculty of Medicine and Dentistry, Universidade de Santiago de Compostela, Spain; 3 Instituto de Investigación Sanitaria de Santiago (IDIS), Santiago de Compostela, Spain; 4 Research Virgen de la Arrixaca Clinical University Hospital, IMIB-Arrixaca, University of Murcia. School of Dentistry, Faculty of Medicine, University of Murcia, Clínica Odontológica Universitaria Hospital Morales Meseguer. Spain; 5 Unit of Dental Pathology and Therapeutics II, School of Medicine and Dentistry, Universidade de Santiago de Compostela, Spain

## Abstract

**Background:**

The objective of this study was to analyse the association between oral and general health variables and obesity indicators with the sensation of dry mouth or xerostomia as evaluated on the Xerostomia Inventory (XI).

**Material and Methods:**

A total of 354 randomly selected subjects participated in this cross-sectional pilot study and completed an anonymous questionnaire. Anthropometric, clinical, and xerostomic variables were evaluated. Kruskal-Wallis, ANOVA and Bonferroni test were used for multiple comparisons. ROC curves and multinomial logistic regression were used to determine the (OR) risk of xerostomia.

**Results:**

A total of 30.7 % of respondents reported xerostomia based on XI. The dry mouth question, the XI taken as a “gold standard”, showed a diagnostic sensitivity of 70.37 %, and a specificity of 83.27 % (AUC=0.768, *p*<0.001). Logistical regression showed the highest xerostomia OR was associated to patients with bad self-perceived health, 6.31 (CI 95% 2.89-13.80, *p*<0.001). In the model adjusted for tooth mobility, bone or respiratory diseases, and the consumption of anxiolytics and antidepressants, the OR was 3.46 (CI 95% 1.47-8.18, *p*=0.005).

**Conclusions:**

a high prevalence of xerostomia was found in this cross-sectional pilot study, which was significantly more frequent in women, and increased with age. Xerostomia was associated to several systemic diseases, psychological conditions, and oral functional disorders such as tooth mobility.

These preliminary results can serve as the basis for developing guidelines for the application of innovative measures designed to improve the quality of life of individuals with xerostomia.

** Key words:**Xerostomia, systemic pathology, oral health, obesity, geriatrics.

## Introduction

Xerostomia is defined as the subjective sensation of a dry mouth and is considered "the illness of modern man". The term is frequently used to refer to a salivary gland dysfunction with decreased salivary secretion, a condition known as hyposialia or hyposalivation. The subjective sensation (xerostomia) and the objective measurement (hyposialia) do not always concur (1). The sensation of oral dryness does not intrinsically involve a diminished quantity of saliva, as some patients diagnosed for hyposalivation do not perceive this sensation. Hyposialia is measured using sialometric (2), and patients are administered the Xerostomia Inventory (XI) to evaluate the severity of the sensation of mouth dryness (3).

The incidence and prevalence of xerostomia is higher in women,and increases with age (4). Xerostomia has been associated to an array of chronic pathologies and medications such as hypertension and Angiotensin-converting enzyme (ACE) inhibitor treatment, diuretics, and medication for psychological disorders such as anxiety or depression (5,6). A significant association has been found between medication and xerostomia, (7) with adverse reactions frequently linked to certain drugs, making mouth dryness a pervasive problem among geriatric patients (8). This segment of the population is numerous in Galician society, where according to the National Institute of Statistics the mean age is around 47.48 years.

The person’s lifestyle pattern such as alcohol consumption and smoking is a key factor both in the occurrence and in long-term xerostomia given that smokers exhibit salivary gland dysfunction with diminished secretion of aqueous (9). In terms of diet, ensuring the daily consumption of fresh fruit and vegeTables and the intake of liquids is vital for rehydration and maintaining oral health (10), given that the correct rehydration of oral mucosa reduces symptoms (11).

Obesity is a major risk factor for an array of diseases,and is related to xerostomia and other oral problems such as caries, periodontitis, and tooth loss (12,13). Dyslipidaemia, arterial hypertension, and hyperglycaemia, together form a cluster of medical conditions disorders broadly referred to as the metabolic syndrome. The underlying causes are multiple and include a sedentary lifestyle, age, genetics, malnutrition, and even masticatory hypofunctionality, as suggested in previous studies of the group, where the number of functional teeth was directly related to abdominal obesity. The increasing prevalence of obesity leads to this metabolic syndrome characterized by abdominal obesity, insulin resistance, hypertension, and dyslipidaemia (14). Previous studies of the group have shown that masticatory hypofunctionality may be associated to this metabolic syndrome, and the number of functional teeth has been directly related to abdominal obesity (15).

The presence of oral problems is associated to a poorer self-perception of quality of life (16). Moreover, bad oral health has been found to raise the risk of certain conditions such as aspiration pneumonia, cardiovascular disorders, diabetes, among others (17), though further research is required in most of the branches of geriatric odontology (18). The objective of this study was to analyse the association between oral and general health variables and obesity indicators with the sensation of dry mouth or xerostomia as evaluated on the XI.

Abbreviations: Body Mass Index (BMI); Waist-to height ratio (WHtR);Xerostomia Inventory (XI).

## Material and Methods

- Study design

This cross-sectional study complied with the ethical principles of the Helsinki Declaration 2013, and was approved by the Ethics Committee of Galicia under reference PGU-HD-2018-01, as part of the research project analysing the association between general and oral health. The data were gathered anonymously from October 2019 to February 2020 in accordance with the recommendations of the STROBE guidelines for observational studies (19). All participants freely volunteered and informed consent was obtained prior to inclusion in the study. Respondents were assured their data would remain anonymous and confidential according to the Spanish Data Protection Laws.

- Questionnaire design

A self-report questionnaire was designed in Google forms, consisting of 36 questions with a binary or multiple response format with closed options. The questionnaire was divided into 5 sections referring to (I) sociodemographic data, (II) oral health, (III) general health, (IV) overweightness and/or obesity, (V) xerostomia. The data was gathered using the questionnaire designed in Google Forms, and was read aloud by the interviewer to assist in the understanding and use of technology when requested by the respondent, or to clarify any queries. I) sociodemographic data: location of questionnaire administration, age, sex, academic status, smoking habits. II) Oral health: tooth mobility, tooth loss, dentures/implants, gingival bleeding, mandibular pain, and dry mouth sensation. III) General Health: hypertension, diabetes, cholesterol, degenerative bone, cardiac, gastrointestinal, and neurological disorders, consumption of anxiolytics and/or antidepressants, physical exercise, good self-perceived general health, consumption of fresh fruit and vegeTables. IV) Overweightness and obesity: height, weight, and waist circumference diameter. V) Xerostomia: XI. The full questionnaire is available in Supplement 1.

- Sample population, inclusion and exclusion criteria

The data were gathered anonymously from a diverse and widespread area of the Autonomous Community of Galicia, north-western Spain: the survey was conducted at streets level, in waiting rooms in health centres and hospitals (among non-odontological healthcare workers), chemists, and service sector workers (in supermarkets, shops, bakeries, and so forth). The sample consisted of 355 adults aged 50 years or older, who were randomly selected from the general population of Galicia. Inclusion criteria: adults aged 50 years or older who volunteered to participate in this study. Exclusion criteria: patients under the age of 50 years, pregnant mothers, respondents refusing consent.

- Variables and data

Xerostomia Inventory (XI): The XI is an 11-item instrument with 5-point graded response format to evaluate the severity aspects of xerostomia as measured on a continuum. The Spanish version (20) of the instrument designed in 1999, has a score range from 0 to 44 points, with high values indicating severe xerostomia symptoms. The value ranges for each degree were as follows: 0-11: no xerostomia, 12-22 mild xerostomia, 23-33 moderate xerostomia, and 34-44 severe xerostomia.

- BMI

The body mass index (BMI) is calculated based on the following formula: bodyweight in kilograms divided by height in meters squared. According to this calculation, the World Health Organisation has classified nutritional status as follows: Very severe thinness ≤ 15; Severe thinness 15-15.9; Thinness 16-18.4; Healthy weight 18.5-24.9; Overweight 25-29.9; Moderate obesity 30-34.9; Severe obesity 35-39.9, and Morbid obesity ≥ 40. As this formula is limited in that it fails to evaluate body fat distribution, the waist-to-height ratio (W/Ht) was also used.

- Waist-to-height ratio (WHtR)

The WHtR has been observed to be a simpler and more predictive indicator than the BMI (21). The waist-to-height ratio (WHtR) is defined as a person’s waist circumference divided by height, both in centimetres. This index is more predictive of the risk of obesity associated to cardiovascular diseases than the BMI. Critical values vary according to age and sex, being 0.6 for over 50 year-olds. Significant gender differences were observed between men and women in the WHtR distribution: extremely thin (men and women <0.34); healthy thin (men 0.35 to 0.42; women 0.35 to 0.41); healthy (men 0.43 to 0.52; women 0.42 to 0.48); overweight (men 0.53 to 0.57; women 0.49 to 0.53); very overweight (men 0.58 to 0.62; women 0.54 to 0.57); morbid obesity (men > 0.63; women > 0.58).

- Statistical analysis

The data were collected and stored on a database specifically designed for this study. Statistical analyses were performed using the SPSS v.24.0 software package (IBM, Statistics, NY, USA). The categorical variables were expressed in frequencies and percentages, and the quantitative variables in means and standard deviation. According to the central limit theorem, the distribution of the quantitative variables could be considered normal as the sample size was large. Contingency Tables were designed to analyse the associations between categorical variables using a chi-squared test, and a Kruskal-Wallis test was used for ordinal-level variables. In order to assess the effects of quantitative variables over qualitative ones, parametric statistics were used for the ANOVA test, and for the Bonferroni post-hoc correction for multiple comparisons. The degree of correlation of certain variables was analysed using Pearson’s correlation coefficient (CC). In order to evaluate the diagnostic efficacy of the XI, ROC curves were used to determine sensitivity, specificity, and the area under the curve (AUC). Both univariate and adjusted multinomial logistic regression were used to determine the (OR) risk of xerostomia. The level of significance was *p* ≤ 0.05.

## Results

The sample consisted of 229 women (64.7 %) and 125 men (35.3%), who were distributed in the following age groups: 269 (75.8%) from 50 to 64 years, 62 (17.5%) from 65 to 79 years, and 24 (6.8%) 80 years or older. The complete data are shown in Table 1. In association to oral health, 45.6 % of respondents had experienced tooth mobility, and 60.2 % lacked dentures or implants. The most prevalent systemic diseases was hypercholesterolemia (32.7 %), followed by degenerative bone conditions (29.3 %), and hypertension in 22.3 % of participants. However, 90.7 % of respondents perceived their general health was good or very good. Moreover, 34.6 % reported no regular physical exercise, and 32.2 % consumed neither fresh fruit nor vegeTables daily. The mean BMI was 25.56 ± 4.40, and the WHtR was 0.56 ± 0.10.

**Figure 1:**
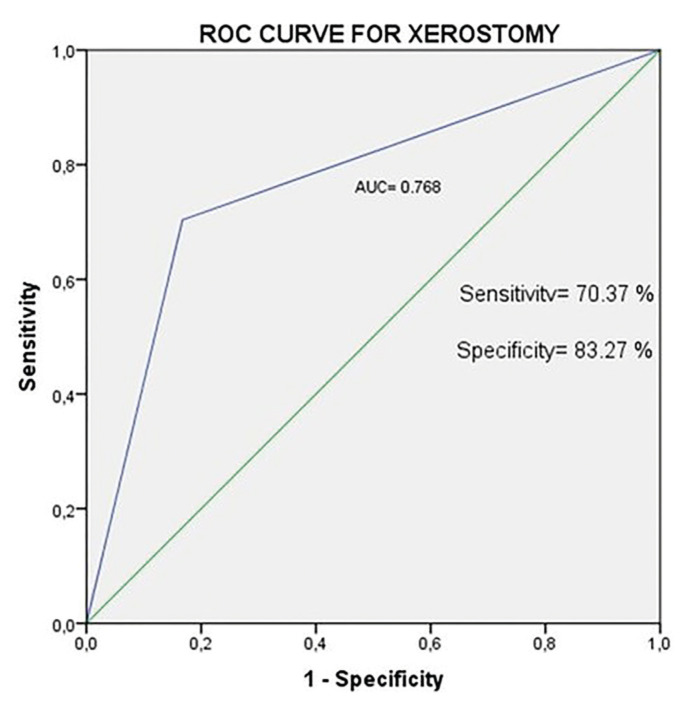
ROC curve for diagnostic yield of the dry mouth question, taken the XI as a “gold standard”: Sensitivity of 70.37 %, and a specificity of 83.27 % (Youden index=0.54, AUC=0.768, *p*<0.001).

As for xerostomia, 33.1 % responded Yes to the question ‘Do you have a dry mouth sensation?’ The mean for the XI test was 9.21 ± 7.70. A total of 69.3 % of respondents reported no xerostomia, 24.5% mild, 5.1 % moderate, and 1.1% severe xerostomia, that is, a total of 30.7 % of those surveyed reported xerostomia. The complete data are shown in Table 2. A correlation coefficient was found between the direct question and the XI test of CC=0.525, *p*<0.001. The dry mouth question, the XI taken as a “gold standard”, showed a diagnostic sensitivity of 70.37 %, and a specificity of 83.27 % (Youden index=0.54, AUC=0.768, *p*<0.001) (Fig. 1).

According to the XI, (24 %) of men reported mild, and (1.6%) moderate xerostomia, whereas 6.6 % of women reported moderate, and 1.7% severe xerostomia (*p*=0.072). Age was a significant factor, the prevalence of xerostomia was highest in the 80-year-old or older age group, with 54.2 % reporting xerostomia as compared to 35.5 % in the 65-79 year-old group, and 27.5 % in the 50-64 year-old group (*p*=0.001). Patients with tooth mobility reported more xerostomia than patients with no tooth mobility, 39.5 % vs. 23.3 % (*p*=0.001).

**Table 1:**
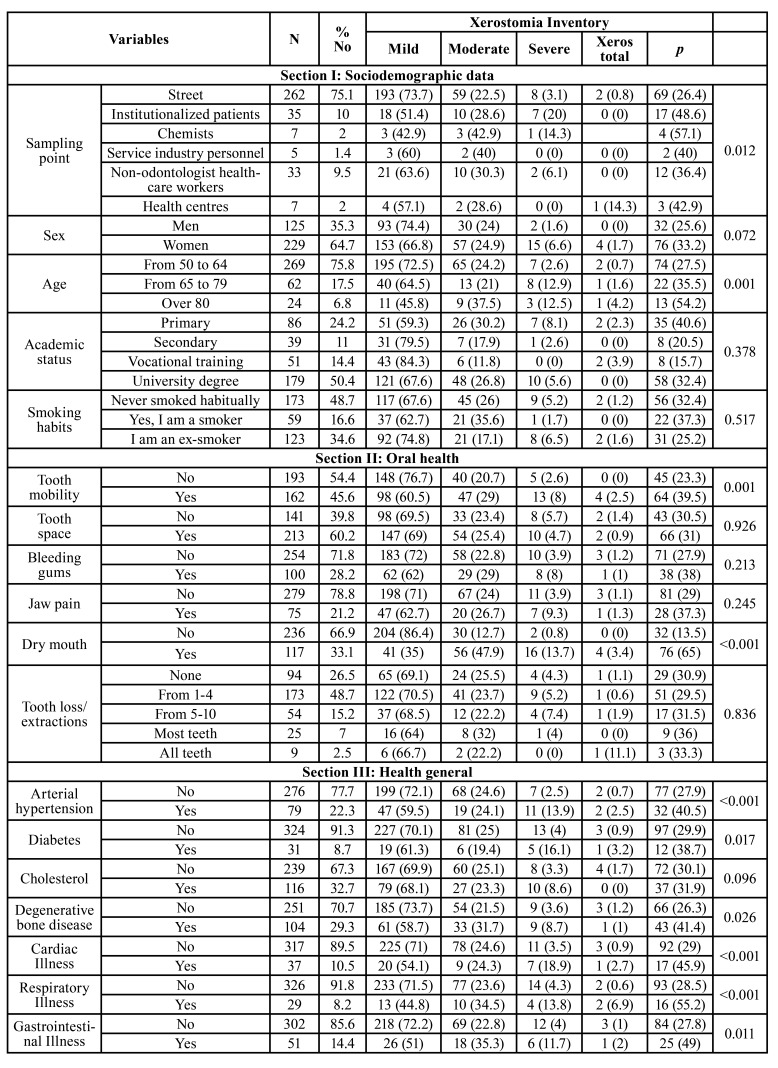
Descriptive sample data and the association of the variables with the Xerostomia Inventory.

**Table 1 cont.**
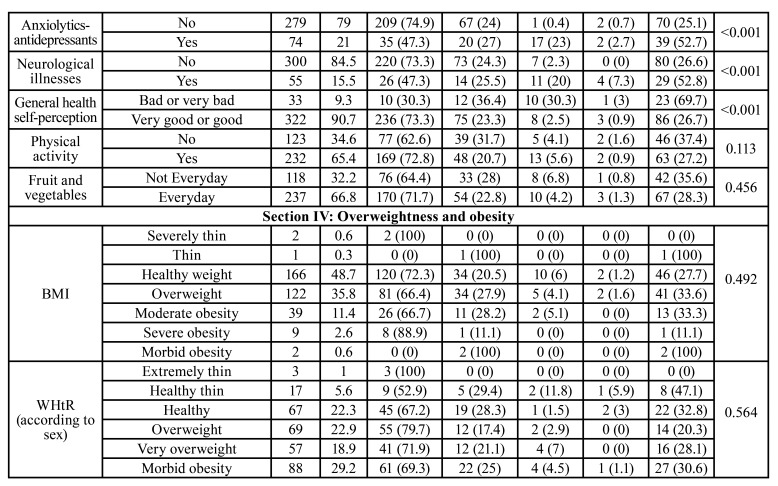
Descriptive sample data and the association of the variables with the Xerostomia Inventory.

**Table 2:**
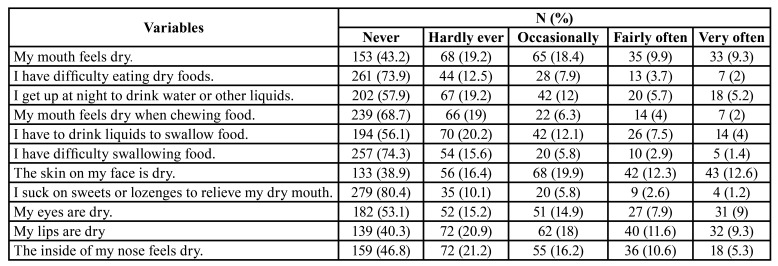
Full data of Xerostomia Inventory.

Statistically significant more xerostomia was reported in patients with arterial hypertension (*p*<0.001), diabetes (*p*=0.017), and bone degenerative (*p*=0.026), cardiac (*p*<0.001), pulmonary (*p*<0.001), or digestive conditions (*p*=0.011). Moreover, significantly more xerostomia was reported in respondents taking anxiolytic and/or antidepressant medication, with a 52.7 % and 42.7 % reporting a certain degree of xerostomia, respectively. Patients reporting good general health exhibited significantly less xerostomia than patients with bad or very bad general health, 26.8 % vs. 69.7 % (*p*<0.001). In this study, neither the BMI nor the WHtR were significantly associated to the presence of xerostomia. However, a statistically significant association was found between the WHtR and the BMI with the number of missing teeth i.e., the more the tooth loss the higher the BMI and WHtR. As for the BMI with no tooth loss, the mean was 24.84 ± 5.0 (CI 95% 23.80-25.88), as compared to the BMI with extensive tooth loss with a mean of 27.86 ± 5.72 (CI 95% 25.44-30.28) (*p*=0.025 Bonferroni test). As for the WHtR with no tooth loss, the mean was 0.53 ± 0.1 (CI 95% 0.51-0.54), in comparison to WHtR with extensive tooth loss with a mean of 0.63 ± 0.1 (CI 95% 0.56-0.69) (*p*=0.003 Bonferroni test).

Logistical regression showed the highest xerostomia OR was associated to patients with bad self-perceived health, 6.31 (CI 95% 2.89-13.80, *p*<0.001). In the model adjusted for tooth mobility, bone or respiratory diseases, and the consumption of anxiolytics and antidepressants, the OR was 3.46 (CI 95% 1.47-8.18, *p*=0.005). Other covariables were statistically significant, but to a lesser extent (Table 3).

**Table 3:**
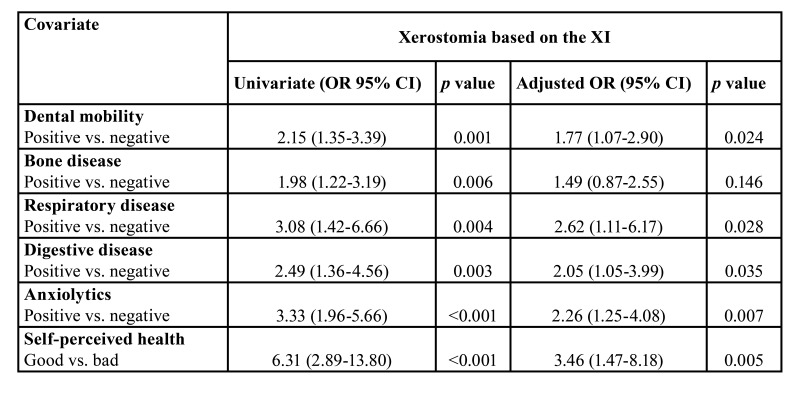
A univariate logistic regression analysis was performed to determine the univariate OR for xerostomia. The adjusted statistical analysis was undertaken using a gradual multivariate logistic regression adjusted for dental mobility, degenerative bone disease, respiratory disease, OR: Ratio of risk.

## Discussion

In this study the prevalence of xerostomia was 30.7% of the sample, almost within the 20 to 30% range observed by other authors (4), with a higher prevalence in women and the elderly. Though the XI data were not correlated to the objective salivation tests of Hijjaw *et al*. (22), regardless of hyposalivation, xerostomia poses a problem per se, as it had an effect on feeling healthy, with xerostomia observed in 69.9 % of respondents in this study reporting bad or very bad self-perceived health. The logistic regression analysis showed an association between the dry mouth sensation and several systemic diseases and psychological disorders. In respondents taking anxiolytic and/or antidepressant medication, the risk of xerostomia was 3.33 higher in the univariate model, and 2.26 in the adjusted model. As regards general health, xerostomia was observed in 40.5% of arterial hypertension patients, which may be due to its influence on the quality and quantity of saliva (23). Moreover, a higher prevalence of xerostomia was observed in diabetic respondents, which agrees with the findings of López-Pintor *et al*. (24). As for study on the association between xerostomia and obesity in Sweden, an association was found in the analysis of raw data, but it disappeared with adjusted confounding factors (25). In this study no significant differences were observed between the BMI and the WHtR in association to xerostomia, but an association was observed between tooth loss and the BMI (*p*=0.025), and the WHtR (*p*=0.003). The results agree with the findings of the meta-analysis published in 2016, where tooth loss was 1.49 times higher in the obese, with a possible bidirectionality between both variables (13).

The mutual and complex association between oral pathology and systemic diseases has been well documented in the literature. Tavares *et al*. observed a correlation between both, with oral health affecting general health and vice versa (26); however, the evidence to date is insufficient to conclusively establish an association (18). Thus, the aim of this study was to obtain an initial snapshot of the association between xerostomia, and oral and systemic health, as well as being the first study to provide data on this association in Spain.

The results of this study corroborated the findings of recent studies that found a xerostomia OR = 5.1 in individuals reporting bad or very bad self-perceived health (27), and substantiated that psychotropic medication is one of the primary causes of the dryness of mouth (6), a view supported by the dryness of mouth sensation OR = 4.74 observed in individuals taking antidepressants. Abdullah *et al*. found patients with systemic pathology presented an OR = 2.80 more xerostomia (28). To our knowledge, this is the first report in the literature describing an association between xerostomia and degenerative bone, cardiac, pulmonary, and digestive illness.

As regards oral pathology, tooth mobility was found to be related to xerostomia (OR of 2.15 in the univariate model, and 1.77 in the adjusted model), but this finding should be validated in a larger sample population. Johansson *et al*. have found an association between xerostomia and several oral problems such as difficulties in chewing, burning and dry mouth sensation, and TMJ pain (27).

Regarding the findings of this study and their clinical implications, the results underscore the importance of correctly diagnosing xerostomia, and the efficacy of questionnaires as diagnostic tools (3). The direct dichotomous question proposed on the dryness of mouth sensation could be of diagnostic utility, with a sensitivity of 70.37 %, taking as a gold standard the cut-off point of 11 on the XI. In this particularly vulnerable group, the diagnosis of certain systemic diseases such as bone, cardiac, or digestive pathologies can assist in the preliminary diagnosis of xerostomia. Correct management can reduce the incidence of oral and systemic pathologies, and improve bad self-perceived health. In terms of health organization, it is recommended the odontologist work in collaboration with health professionals in other disciplines such as primary care, traumatology, and cardiology, in order to develop a coordinated and multidisciplinary approach to dental health care programs.

The interpretation of the results of this study are subject to several limitations, given that the data was circumscribed to only one autonomous community in Spain, it is not entirely epidemiologically representative at a national level. Moreover, the cross-sectional design cannot prove causality, that would be ideal for converting into clinical observation, which underscores the needs for prospective longitudinal studies. Notwithstanding, this study has noTable strengths, the proper calibration of the researchers, participants, and the selection of diagnostic criteria reasonably. As for the generalization of the results on the basis of the experimental design, the results can be extrapolated to other contexts bearing in mind the inter-territorial homogeneity

In conclusion, a high prevalence of xerostomia was found in this cross-sectional pilot study, which was significantly more frequent in women, and increased with age. Xerostomia was associated to several systemic diseases (such as arterial hypertension, diabetes, and bone, heart, lung, and digestive diseases), psychological conditions, and oral functional disorders such as tooth mobility. These preliminary results can serve as the basis for developing guidelines for the application of innovative measures designed to improve the quality of life of individuals with xerostomia. The comorbidity between xerostomia, deteriorated general health, and oral health disorders should be appraised in the dental management of elderly patients. The diagnosis of associated diseases and their medications can assist the odontologist in the preliminary diagnosis. Further studies are required to validate the conclusions of this study.
